# An analysis of blood pressure measurement in a primary care hospital in Swaziland

**DOI:** 10.4102/phcfm.v6i1.590

**Published:** 2014-12-09

**Authors:** Ganizani Mlawanda, Michael Pather, Srini Govender

**Affiliations:** 1 Faculty of Medicine and Health Sciences, Department of Interdisciplinary Health Sciences, Division of Family Medicine and Primary Care, University of Stellenbosch, Tygerberg, South Africa; 2 Royal Swaziland Sugar Corporation (RSSC) Medical Services, Mhlume, Swaziland; 3 Khayelitsha Community Health Centre, Khayelitsha, South Africa

## Abstract

**Background:**

Measurement of blood pressure (BP) is done poorly because of both human and machine errors.

**Aim:**

To assess the difference between BP recorded in a pragmatic way and that recorded using standard guidelines; to assess differences between wrist- and mercury sphygmomanometer-based readings; and to assess the impact on clinical decision-making.

**Setting:**

Royal Swaziland Sugar Corporation Mhlume hospital, Swaziland.

**Method:**

After obtaining consent, BP was measured in a pragmatic way by a nurse practitioner who made treatment decisions. Thereafter, patients had their BP re-assessed using standard guidelines by mercury (gold standard) and wrist sphygmomanometer.

**Results:**

The prevalence of hypertension was 25%. The mean systolic BP was 143 mmHg (pragmatic) and 133 mmHg (standard) using a mercury sphygmomanometer; and 140 mmHg for standard BP assessed using wrist device. The mean diastolic BP was 90 mmHg, 87 mmHg and 91 mmHg for pragmatic, standard mercury and wrist, respectively. Bland Altman analyses showed that pragmatic and standard BP measurements were different and could not be interchanged clinically. Treatment decisions between those based on pragmatic BP and standard BP agreed in 83.3% of cases, whilst 16.7% of participants had their treatment outcomes misclassified. A total of 19.5% of patients were started erroneously on anti-hypertensive therapy based on pragmatic BP.

**Conclusion:**

Clinicians need to revert to basic good clinical practice and measure BP more accurately in order to avoid unnecessary additional costs and morbidity associated with incorrect treatment resulting from disease misclassification. Contrary to existing research, wrist devices need to be used with caution.

## Abstrak

**Une analyse de la mesure de la tension artérielle dans un hôpital de soins primaires au Swaziland**.

**Contexte:**

La tension artérielle (BP) est mal mesurée à cause de l'erreur humaine et des machines.

**Objectif:**

Evaluer la différence entre la BP mesurée d'une façon pragmatique et celle mesurée à l'aide des directives classiques; pour évaluer les différences entre les mesures prises au tensiomètre de poignet et celles prises au tensiomètre à colonne de mercure; et pour évaluer l'impact sur la prise de décision clinique.

**Cadre:**

L'hôpital Mhlume de la Royal Swaziland Sugar Corporation, au Swaziland.

**Méthode:**

Après avoir obtenu le consentement, la BP a été mesurée d'une façon pragmatique par une infirmière qui a décidé du traitement. Ensuite, on a remesuré la BP des patients avec le tensiomètre à colonne de mercure et le tensiomètre de poignet.

**Résultats:**

La prévalence de l'hypertension était de 25%. La tension artérielle systolique moyenne était 143 mmHg (pragmatique) et 133 mmHg (standard) avec un tensiomètre à mercure et 140 mmHg pour la BP standard évaluée avec un tensiomètre de poignet. La BP diastolique moyenne était 90 mmHg, 87 mmHg et 91 mmHg respectivement pour la mesure pragmatique, la standard au mercure et celle au poignet. Les analyses de Bland Altman ont montré que les mesures de BP pragmatiques et standard étaient différentes et ne pouvaient pas être cliniquement interverties. Les décisions de traitement entre les mesures basées sur la BP pragmatique et la BP standard correspondaient dans 83.3% des cas, alors que les résultats des traitements de 16.7% des participants étaient mal classés. 19.5% des patients avaient été mis sous un traitement erroné antihypertenseur basé sur la BP pragmatique.

**Conclusion:**

Les cliniciens doivent revenir aux bonne pratiques cliniques de base et mesurer la BP avec plus de précision afin d’éviter les coûts supplémentaires superflus et la morbidité liée au traitement incorrect dû à la mauvaise classification de la maladie. Contrairement aux recherches existantes, les tensiomètres de poignet doivent être utilisés avec précaution.

## Introduction

Hypertension is a consistent, powerful and independent risk factor for cardiovascular disease, stroke and renal disease.^1^ Diagnosis of hypertension is based on measurement of blood pressure (BP). Obtaining accurate BP readings has been noted to be a challenge faced by health professionals at all levels.^2^ A large number of surveys have shown that physicians, along with other healthcare providers, seldom follow established guidelines for measurement of BP.^3^ This study analysed variations between pragmatic (‘real-life’) and standardised (as per protocol) BP measurement. Technology has brought in various BP measuring devices, a common one in primary care being the wrist sphygmomanometer as opposed to the ‘gold standard’, but environmentally unfriendly, mercury sphygmomanometer. How does BP measurement from wrist device compare with the gold standard?

### Literature review

Hypertension is a common health burden affecting both developed and developing nations.^4^ The prevalence of high BP increases dramatically with age, with the lifetime risk of high BP approaching 100%.^5^ Extensive data have shown beyond doubt the benefit of controlling hypertension.^6^

Control of BP begins with accurate measurement that leads to appropriate diagnosis, assessment of cardiovascular risk and treatment decisions.^1–6^ The target BP for patients using anti-hypertensive treatment has been lowered for those with diabetes or renal disease,^1^ thus, it has become increasingly important to be able to detect small differences in BP. Whilst BP measurement is a vital clinical skill, it is performed poorly by all categories of healthcare professional.^4^ There are, in general, three sources of error in the indirect measurement of BP: (1) observer bias; (2) faulty equipment; and (3) failure on the part of clinicians to standardise the measurement techniques.^7^

The mercury sphygmomanometer, because of its accuracy and reliability, is widely regarded as being the gold standard against which all other devices for BP measurement should be compared.^5^ As a result of environmental awareness, there has been increasing pressure to remove medical devices containing mercury from clinical areas, which is leading to the gradual decline in use of the mercury sphygmomanometer and, as a result, automated BP devices have been adopted by clinicians for their convenience and ease of use.^8^

Rose suggested that the observer was the most critical component of accurate BP measurement.^9^ Petrie et al. declared that only an observer who is aware of the factors that lead to false readings should measure BP, because ‘wrong readings obtained through failure to use the proper technique often lead to the wrong diagnosis, which may result in unnecessary or inappropriate treatment and follow up’.^10^

In a study by Roubsanthisuk, Wongsurin and Saravich, physicians and trained nurses were compared, showing that trained nurses overestimated, rather than underestimated, blood pressure, but systolic BP underestimation was very common in participants with moderate to severe hypertension.^11^ ‘Systolic BP underestimation of > 5 mmHg was as high as 57.5% by trained nurses [using the traditional device] versus 33.8% by the automatic device, indicating that nurses tended to underestimate BP in participants with more severe hypertension’.^11^ The BP measurements done by nurses were found to be consistently higher than those recorded by doctors.^11^ McKay et al. noted that few physicians ask their patients to rest for at least five minutes before BP measurement as recommended and, as a consequence, BP done by doctors was consistently high because of the ‘white-coat’ effect.^12^ Contrary to the recommended five minutes of rest, it appears that 10 minutes rest before clinic BP evaluation could improve further the precision and accuracy of the measurement and implies that the optimal time at rest before clinic BP measurement is still undefined.^13^

Clinicians should also be aware that BP in human beings is affected by multiple stimuli, such as respiration, temperature, body posture, emotional or physical stress, meals, alcohol, or caffeine and smoking and hence these factors should be taken into consideration during measurement of BP.^14^ For some patients, BP measurements taken in a doctor's rooms may not be an accurate representation of their typical BP. In up to 25% of patients, this measurement is higher than their typical BP – a phenomenon known as ‘white-coat hypertension’.^14^

From the literature reviewed, it is clear that BP measurement is subject to errors, thus there are still some social and scientific questions which need clarity and further research, especially in resource-limited settings. Literature review concluded that with proper measurement technique, machine variation between the gold standard mercury sphygmomanometer and the wrist is minimal.^3, 7, 10^ In addition there are problems associated with pragmatic-nature BP measurement and other observer-related errors.^1–10^

Nearly all the articles found on literature review are from developed countries with a good patient-to-health-worker ratio. In a developing country setting, where the patient-to-health-worker ratio is low and resources limited, the potential for BP measurement errors may be worse. One obvious question was on assessment of the reliability of BP measurement methods, looking at both sphygmomanometer and observer differences in resource-limited settings. In so doing, such research will further enlighten health workers about the trustworthiness of BP readings and ensure that health workers are treating BP optimally. Problems related to over- or under-treatment may be serious and, if identified early, could reduce unnecessary morbidity and mortality. Most of the prior studies have focused mainly on sphygmomanometer-related differences.

### Study rationale and motivation

An analysis of variations between pragmatic or ‘real-life’ and standard BP measurement based on the ‘gold standard’ would be useful in improving chronic disease management and ensuring effective use of already-strained resources in primary care. This study will have an impact on increasing awareness of human-induced variation in BP measurement and its impact on therapeutic decisions; hence, it may motivate clinicians to follow protocol. In the long run, it may have some economic advantages in saving cost of drugs erroneously prescribed to those who, if BP had been recorded properly, would not need treatment.

### Aims and objectives

#### Research question

Is there a difference between pragmatic and standard BP measurement in primary care?

#### Aims

1. To ascertain variations between standard and pragmatic BP measurements and comparison of wrist BP and mercury sphygmomanometer-based BP.
2. To assess the impact of any differences on treatment decision.

#### Objectives

1. To quantify the existence of any differences between BP recorded in a pragmatic way and that recorded using standard BP measurement protocols.
2. To quantify any discrepancy between BP measurements done by wrist sphygmomanometer when compared to mercury sphygmomanometers.
3. To assess if the differences in BP measurement have impact on treatment decisions: whether or not to treat, to start anti-hypertensive treatment or to adjust hypertension treatment.

## Research methods and design

### Study design

A cross-sectional study design was used.

### Study setting

This study was done at Royal Swaziland Sugar Corporation (RSSC) Mhlume hospital, targeting outpatients. RSSC Mhlume hospital is a rural primary care facility in the eastern part of Swaziland The facility has a turnover of about 5000 patients per month and offers mostly primary care with minor office procedures. It serves a catchment area of about 30 000.

### Study population

The study population comprised adult (> 18 years) patients, with or without hypertension, who accessed primary care at the RSSC hospital during the study period June 2011 to December 2011 and who gave consent to participate in the study.

### Sample size and sampling method

Every fourth patient who had attended the outpatient clinic was eligible for selection. A sample size of 60 was used, based on statistical calculations and sample size from similar studies.^15^ Statistically, two observations per subject achieves an 80% power to detect an intra-class correlation difference of 0.15 using an *F*-test with a significance level of 0.05. In a similar study of agreement, Bland recommends a sample size of 30 as a ‘good sample’ and 60 as ‘excellent’, as it gives a 95% confidence interval of +/-0.34s, where s is the standard deviation of the differences between measurements by the two methods.^15^

### Data collection and measurement method

Informed consent was obtained from eligible patients. Participants had BP assessed in a pragmatic way by nurse practitioners who would give their therapeutic decision based on their readings. Participants had BP re-assessed according to the standard protocol, using mercury sphygmomanometer and wrist sphygmomanometer alternately. To reduce bias, the order of measurement for pragmatic or standard BP measurements was alternated for successive patients. Finally, demographic and relevant clinical data were collected into a ‘Data Collection’ form, which was subsequently entered into a Microsoft^®^ Excel spreadsheet for analysis.

### Reduction of bias

To improve internal validity, the potential biases were handled as laid out below.

#### Selection bias

For the reduction of selection bias, a systematic random sample (every fourth patient) was used.

#### Measurement bias

This level of bias could occur at any stage during the measurement, recording, management or analysis of the data. Notable biases were the Hawthorne effect (nurses could change their BP measurement routine because they were aware of the investigation underway) and observer diagnostic suspicion bias. These were reduced by blinding the nurse researcher to results from the nurse practitioners and nurse practitioners were blinded to the ongoing study. Use of validated, standardised and calibrated sphygmomanometers reduced instrument variation. Batteries for the wrist devices were replaced regularly. To reduce subject physiologic variation, as well as the known regression to mean with repeated BP measurement phenomenon,^16^ the standard BP was measured within a few minutes before or after the pragmatic BP.

#### Confounding

Time between performing the BP measurements was an important confounder. Blood pressure tends to come down with time, which is known as regression to the mean. The time between pragmatic and standard BP assessment was kept at a minimum so as to reduce the possibility of confounding bias. Previous studies indicate that a time lag of less than 10 minutes does not have any significant effect on the BP result.^13^

### Data/statistical analysis

Microsoft^®^ Excel was used to capture the data and the data analysis software system, STATISTICA version 9 (StatSoft Inc., 2009), was used to analyse the data. The statistical analysis comprised both descriptive and analytical statistics. For descriptive statistics, summary statistics were used to describe the variables. The Wilcoxon sign rank test was used to assess differences between means of BP. For analytical statistics, simple logistic regression, Pearson correlation (*r*), intra-class correlation coefficient (ICC) and Kappa were used appropriately. Standard reference scales were used for Pearson, ICC and Kappa. The Bland and Altman (BA) method of analysis of agreement was used for further assessment of agreement. Reference ranges for comparison of BA analysis were within 10 mmHg for diastolic BP and within 20 mmHg for systolic BP, because these are known ranges for hypertension severity grading.^4–6^ Throughout the analysis, a *p*-value of *p* < 0.05 represented statistical significance in hypothesis testing and 95% confidence intervals were used to describe the estimation of unknown parameters.

### Ethical considerations

Ethical approval for the study was granted by the University of Stellenbosch Human Research Ethics Committee (reference number N10/11/394) on 13 May 2011. Institutional ethical approval was also obtained.

## Results

Sixty outpatients consented to participate in the study, of which 32 were men. The mean age of the participants was 42.6 years, the mean weight 77.8 kg and the mean height 1.6 metres. The prevalence of hypertension was 25%. Twenty-eight per cent of the participants had co-morbid diseases.

The mean systolic BP was 143 mmHg for pragmatic BP, 133 mmHg for standard BP using mercury sphygmomanometer and 140 mmHg for standard BP assessed using wrist device. The mean diastolic BPs were 90 mmHg, 87 mmHg and 91 mmHg for pragmatic, standard mercury and wrist, respectively. It took an average of 4.2 minutes between pragmatic and standard BP measurement.

Three participants reported either having a full bladder or having eaten within 30 minutes before BP assessment, five had exercised, one had smoked and taken coffee and seven reported some degree of psychological stress. Table 1 summarises the findings.

**TABLE 1a:** Demographic characteristics of participants.

Characteristics	Variables	s.d.
Males (*n*)	32	-
Females (*n*)	28	-
Mean age in years (standard deviation)	43	14.2
Mean weight in kilograms (standard deviation)	78	19.4
Mean height in centimetres (standard deviation)	164	8.5
Mean body mass index	29	-
Prevalence of hypertension	25%	-
Co-morbid conditions	28%	-
Mid-upper arm circumference (in centimetres)	32	-

**TABLE 1b:** **C**linical characteristics of participants.

Treatment decision	No treatment		Treat		Change treatment	
		
	*n*	%	*n*	%	*n*	%
Based on pragmatic blood pressure	32	53	18	30	10	17
Based on standard blood pressure	41	68	11	18	8	13

**TABLE 1c:** Clinical characteristics of participants.

Blood pressure	Method	Observation	Mean	25th percentile	50th percentile	75th percentile
Systolic blood pressure in mmHg	Pragmatic	60	143	120	140	163
	Standard using mercury device	60	133	110	130	151
	Standard using wrist device	60	140	123	138	155
Diastolic blood pressure in mmHg	Pragmatic	60	90	73	90	105
	Standard using mercury device	60	87	75	85	102
	Standard using wrist device	60	91	77	86	106
	Mean time between pragmatic and standard blood pressure measurements (minutes)	4	-	-	-	-

### Analytical statistical results

The Pearson correlation coefficient (*r*) was the same, 0.9, for systolic and diastolic BP for all BP methods which were being compared, corresponding to ‘good association’ between pairs being compared. The ICC (model 2) was consistent with ‘almost perfect agreement’ for all methods compared. Thus *r* and ICC could not differentiate further the level of agreement between the methods in study. Adjustment for confounding was done: neither psychological stress, full bladder, eating a meal, exercise, smoking nor taking coffee within 30 minutes before BP assessment were confounding factors based on less than 10% difference of *r*, ICC, Kappa and BA results. The key results are presented in Tables 2 and 3.

**TABLE 2:** Pearson (*r*), Intra-class coefficient (ICC) and regression equations for blood pressure (BP) measurement methods.

Blood pressure	Methods in comparison	Pearson coefficient, *r* (* interpretation)	Intra-class coefficient († interpretation)	Regression equations for relationship between blood pressure methods (‡ interpretation)
Systolic blood pressure	Standard/pragmatic	0.9 (good association)	0.8 (almost perfect)	SBPMc = −10.7 + 1.2 SBPPr (gradient 1.2; intercept 10.7)
	Standard/wrist	0.9 (good association)	0.9 (almost perfect)	SBPMc = 20 + 0.8 SBPWr (gradient 0.8; intercept 20)
	Pragmatic/wrist	0.9 (good association)	0.9 (almost perfect)	SBPPr = −2.5 + 1.0 SBPWr (gradient 1.0; intercept −2.5)
Diastolic blood pressure	Standard/pragmatic	0.9 (good association)	0.9 (almost perfect)	DBPPr = −0.7 + 1.0 DBPMc (gradient 1; intercept −0.7)
	Standard/wrist	0.9 (good association)	0.9 (almost perfect)	DBPMc = 10.6 + 0.8 DBPWr (gradient 0.8; intercept 10.6)
	Pragmatic/wrist	0.9 (good association)	0.9 (almost perfect)	DBPPr = 2.5 + 1.0 DBPWr (gradient 1; intercept 2.5)

* Interpretation based on: −1.0 to −0.7 strong negative association; −0.7 to −0.3 weak negative association; −0.3 to +0.3 little or no association; +0.3 to +0.7 weak positive association; +0.7 to +1.0 strong positive association.

† Interpretation based on: ICC can be interpreted as follows: 0–0.2 indicates poor agreement: 0.3–0.4 indicates fair agreement; 0.5–0.6 indicates moderate agreement; 0.7–0.8 indicates strong agreement; and > 0.8 indicates almost perfect agreement.

‡ Abbreviations: SBPMc, Sytolic BP Mercury; SBPPr, systolic BP Pragmatic; SBPWr, systolic BP wrist; DBPPr, diastolic BP Pragmatic; DBPMc, diastolic BP Mercury; DBPWr, diastolic BP wrist.

**TABLE 3:** Bland Altman analyses: Results and interpretation.

Blood pressure	Measurements	Limits of agreement between methods under study			Do the methods agree clinically?

		Bias (95% CI)	Lower (95% CI)	Upper (95% CI)	
Systolic blood pressure	Pragmatic/ideal	-9.6 (-13.2 to -6.1)	-36.6 (-42.7 to -30.5)	17.4 (11.2 to 23.5)	No
	Wrist/ideal	7.1 (4.1 to 10.0)	-15.4 (-20.5 to -10.3)	29.6 (24.5 to 34.7)	No
	Pragmatic/wrist	-2.6 (-5.8 to 0.7)	-26.9 (-32.4 to -21.4)	21.8 (16.3 to 27.3)	No
Diastolic blood pressure	Pragmatic/ideal	-3.0 (-5.6 to -0.4)	-22.6 (-27.0 to -18.1)	16.6 (12.1 to 21.0)	No
	Wrist/ideal	3.7 (1.6 to 5.7)	-11.7 (-15.1 to -8.2)	19.0 (15.5 to 22.5)	No
	Pragmatic/wrist	0.7 (-1.8 to 3.2)	-18.4 (-22.7 to -14.1)	19.8 (15.4 to 24.1)	No

CI, confidence interval.

Interpretation based on comparison of limits of agreement to clinically-acceptable range of blood pressure (BP), within 10 mmHg for diastolic BP and 20 mmHg for systolic BP.

#### Comparison of pragmatic and standard blood pressure

**FIGURE 1:**
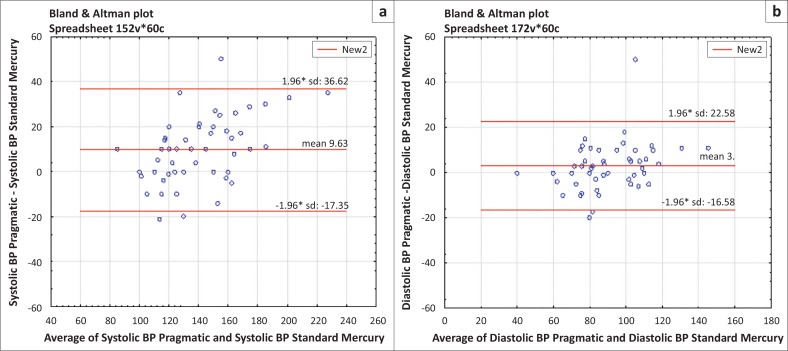
Bland Altman plot for (a) systolic and (b) diastolic blood pressure (BP): standard mercury compared with pragmatic BP.

For systolic BP, the regression relationship was summarised as SBPMc (systolic BP, mercury) = –10.7 + 1.2 SBPPr (systolic BP, pragmatic). For agreement, the bias was 9.6 mmHg with limits of agreement of –17.4 mmHg to 36.6 mmHg. Using the bias alone, 9.6 mmHg, this would equate to excellent clinical inter-changeability based on a clinically-significant BP range of within 20 mmHg. However, the limits of agreement were too wide for the two methods to be regarded as agreeing clinically. Figure 1 illustrates the distribution on a BA plot. For diastolic BP, the regression equation DBPPr (diastolic BP, pragmatic) = –0.7 + 1.0 DBPMc (diastolic BP, mercury) summarised the relationship of diastolic BP between pragmatic and standard mercury-based BPs. The BA bias of 3.0 could have meant excellent agreement but the limits of agreement were again too wide (−16.6 mmHg to 22.6 mmHg) for agreement based on comparison to a clinically-interchangeable BP range of within 10 mmHg.

#### Comparison of wrist and mercury blood pressure

**FIGURE 2:**
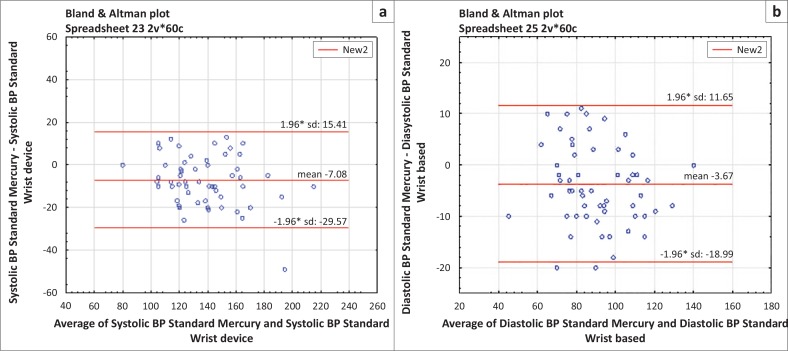
Bland Altman plot for (a) systolic and (b) diastolic blood pressure (BP): standard mercury compared with wrist BP.

For systolic BP, the corresponding regression equation was SBPMc = −2.5 + 1.0 SBPWr (systolic BP, wrist). The BA analysis showed a bias of 7.1 mmHg and limits of agreement, −15.4 mmHg (lower) and 29.6 mmHg (upper), which were outside the clinical reference range for inter-changeability, within 20 mmHg. For diastolic regression the equation was linear, with DBPMc = 10.6 + 0.7 DBPWr (diastolic BP, wrist), a sign of good positive association. The limits of agreement, −19.0 mmHg (lower) to 11.7 mmHg (upper), confirmed poor clinical agreement when compared to the clinically-acceptable range of agreement, within 10 mmHg. Figure 2 illustrates the regression line and BA plots.

#### Comparison of wrist and pragmatic blood pressure

**FIGURE 3:**
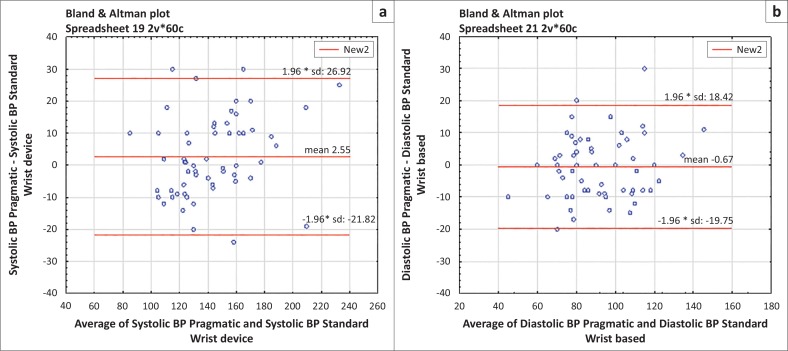
Bland Altman plot for (a) systolic and (b) diastolic blood pressure (BP): standard wrist compared with pragmatic BP.

Finally, pragmatic BP and wrist-based standard BP were also compared for completeness. For systolic BP, *r* had a positive association. The BA plot in Figure 3 shows that the two methods could not be used interchangeably because the limits of agreement were wider than the within-20 mmHg clinical reference range. Similarly, for diastolic BP, the limits of agreement precluded exchangeable use as they were outside the within-10 mmHg clinical reference range.

### Comparison of treatment decisions

Scores for treatment decisions (whether to start anti-hypertensive = 1; alter anti-hypertensive treatment = 2; or defer treatment = 0) were subsequently compared between decisions based on pragmatic BP and those based on standard mercury-based BP. The Kappa score was 0.7 which equates to ‘good agreement’ based on the widely-accepted Byrt's criteria (see Note under B). Overall (without stratifying), the treatment outcomes concurred in 83.8% of the cases, hence 16.7% were misclassified when compared with the standard BP. For the decision not to start treatment 78% of instances concurred; for the decision to start treatment, 90.9% agreed; and for the decision to adjust treatment, the agreement was 100%. Of the patients who were not supposed to start treatment (basing on the standard mercury-based BP), 19.5% (*n* = 8/41), were classified erroneously as requiring anti-hypertensive therapy when using pragmatic BP. Of those who needed to change treatment, the two BPs concurred (100%). Table 4 summarises the overall agreement level and Box 1 gives the stratified treatment outcomes.

**TABLE 4:** Comparison of treatment decisions between pragmatic and standard blood pressure measurements.

Agreement	Expected agreement	Kappa	Lower 95% CI	Upper 95% CI	*p*-value*
83.8	44.2	0.7	0.5	0.9	0

CI, confidence interval.

* Byrt criteria: Excellent agreement 0.93–1.00; very good agreement 0.81–0.92; good agreement 0.61–0.80; fair agreement 0.41–0.60; slight agreement 0.21–0.40; poor agreement 0.01–0.20; no agreement ≤ 0.00.

**BOX 1:** Contingency table for per-stratum treatment outcomes comparing pragmatic to standard blood pressure.

		Treatment plan based on standard mercury blood pressure			
		0	1	2	Total
**Treatment plan based on pragmatic blood pressure**	**0**	32	0	0	***32***
		78.10%	0%	0%	
	**1**	8	10	0	***18***
		19.50%	90.90%	0%	
	**2**	1	1	8	***10***
		2.40%	9.10%	100.00%	
	**Total**	***41***	***11***	***8***	***60***

0, no treatment; 1, treatment; 2, treatment changed.

## Discussion

With hypertension defined as BP 140/90 mmHg, one in five (20%) South Africans have hypertension,^4^ a prevalence which was lower than the 25% from this study. Since hypertension is more common in black people,^4^ the higher prevalence was most likely a result of the black-ethnic predominance of the study population.

The next step was a comparison of pragmatic and standard BP measurements. Health workers generally do not follow BP measurement guidelines. In a study on the BP measurement behaviour of clinicians done by Villegas et al., none of the physicians tested followed all the recommendations of the American Heart Association when measuring BP and a few recommendations were only followed by a minority of the physicians studied.^17^ In this study, there was no clinical agreement between pragmatic and standard BP measurements for both systolic and diastolic BP. Pragmatic systolic BP was at least 10.7 mmHg higher than standard mercury BP. For diastolic BP, pragmatic readings were at least 3 mmHg higher than the standard mercury readings. These results were similar to those from a study by Myers et al. which found that when the primary care physician recorded BP using a mercury or anaeroid device, the resulting value frequently tended to be higher than what it would be if measurement guidelines were adhered to strictly.^18^ Similarly Campbell and McKay concluded that pragmatic readings, namely, those obtained with little regard for patient factors or recommended technique, cause errors in BP assessment and do not correlate effectively with target organ damage; as such, no evidence exists to support the use of pragmatic readings in assessing a patient's need for pharmacologic treatment.^19^ However, standardised readings, namely, those that follow recommended protocols, demonstrate high correlation with hypertensive target organ damage and were used in the major randomised controlled trials that showed the benefits of pharmacotherapy.^19^

The clinical consequences of poor BP measurement are well documented in literature: consistent overestimation of diastolic BP by as little as 5 mmHg may more than double the number of patients with hypertension in a physician's practice.^20^ People who are identified incorrectly as having hypertension may experience adverse effects of medication and have increased medical insurance and treatment costs.^21^ Conversely, consistent underestimation of diastolic pressure by the same margin would reduce by 62% the number of patients perceived as being hypertensive.^21^ These errors could deprive patients of therapy which has been proven to be beneficial, thus leading to possible increases in serious medical and social complications.^21^

In this study, 19.5% of patients who were started on anti-hypertensive therapy based on pragmatic BP actually did not need any treatment. This trend was similar to many studies which showed increased diagnosis of hypertension if BP was not measured according to guidelines.^5, 6, 10, 11, 17, 19, 20^ Overall, 16.7% of participants had their treatment outcomes misclassified. Of those who needed treatment, there was a concordance of 91% between pragmatic and standard BP-based decisions. However, for those hypertensive patients who needed to have their treatment adjusted, pragmatic and standard BP had 100% concordance. The likely explanation for this is that when BP was markedly elevated, there was no difference between pragmatic and standard BP.

Comparison of wrist and mercury BP measurements was subsequently performed. Standard mercury diastolic and systolic BPs were consistently higher when using a wrist device. For systolic BP, the difference was as much as 20 mmHg, whilst it was approximately 10 mmHg for diastolic BP, a sharp contrast to previous studies which found similarities between mercury and wrist devices.^3, 7, 10, 22^ We suspected that the difference was mostly because of the precise arm position and a known problematic phenomenon of wrist devices in which there is a systematic error introduced by the hydrostatic effect of differences in the position of the wrist relative to the heart.^22^ This can be avoided if the wrist is always at heart level when the readings are taken, but there is no way of knowing retrospectively whether this was performed when a series of readings are reviewed.^22^

The mercury sphygmomanometer is generally regarded as the gold standard against which all other devices for BP measurement should be compared.^5^ However, recent studies have shown that ambulatory BP measurements correlate better with the exact BP. Hodgkinson et al. have recently concluded that ambulatory BP was more cost effective than clinic or home BP.^23^ However, guidelines for diagnosis and treatment of hypertension are still based on clinic BP measurements.^4, 10^

Finally, a statistical lesson! Statistical methods for comparison methods have been subject of discussion amongst clinicians. The BA method is regarded as the gold standard.^23^ Several papers have challenged the shortfalls of BA analysis,^24^ but Bland and Altman have stated that the use of correlation coefficients is wrong for these types of studies.^25^ In this study, intra-class correlation, Pearson's coefficient and linear regression both fell short of explicitly analysing the research question.

### Strengths and weaknesses of the study

The main strength was that this study design was fast and inexpensive and was done in a resource-limited setting approximating most third-world institutions. It gave a useful initial overview of the problem, including the community prevalence. The statistical methods used were appropriate for studies of this nature.

There was very limited potential to make causal inference of any differences, an obvious weakness of this study. Secondly, we could not claim success with minimising regression to mean with the serial BP measurements as the exact time to ensure that regression to mean is rectified, is unknown. In addition, it was impossible to totally eliminate observer bias despite ‘blinding’ the nurses as there was always room for discussion when they meet outside the study centre; hence, the ‘pragmatic’ BPs might not have been as pragmatic as we expected. The other potential confounder was that the pragmatic BP was done by different nurse practitioner. No adjustments were made for this as surely their BP measurement techniques would differ. The other problem was diagnostic on the part of nurses: the clinical decision to start treatment. Usually, a number of readings are required to start treatment unless there are risk factors, significant target organ damage or BP was markedly elevated. The nurse practitioners might have over-diagnosed hypertension as they relied erroneously on one reading, even when BP was mildly elevated.

### What is already known on this topic?

There are differences between pragmatic and standard BP but wrist and mercury BP readings are usually comparable.

### What this study adds

This study further confirmed the existence of differences between pragmatic and standard BP measurements in a resource-limited setting. The difference leads to 16.7% disease status misclassification. Wrist and mercury devices potentially lead to conflicting results, which is contrary to earlier studies. Pearson and Intra-class correlation coefficients are weak statistical methods in studies of this nature.

## Conclusion

There is a difference between pragmatic and standard BP measurements which affect the decision to start treatment and the decision to initiate treatment, but not the decision regarding alteration of regime for those already on treatment. There are also marked differences between wrist- and standard mercury-based BP devices which also affect treatment decision-making. In future, when assessing agreement between clinical methods, the BA method is more conclusive than correlation coefficients. Clinicians need to revert to basic good practice and measure BP more accurately so as to avoid unnecessary additional costs and morbidity associated with incorrect treatment resulting from disease misclassification. Wrist devices need to be used with caution.

## References

[CIT0001] Neaton JD, Wentworth D Serum cholesterol, blood pressure, cigarette smoking, and death from coronary heart disease. Overall findings and differences by age for 316,099 white men. Multiple Risk Factor Intervention Trial Research Group. Arch Intern Med. 1992;152(1):56–64. http://dx.doi.org/10.1001/archinte.1992.00400130082009 1728930

[CIT0002] Kim JW, Bosworth HB, Voils CI, et al. How well do clinic-based blood pressure measurements agree with the mercury standard? J Gen Intern Med. 2005;20(7):647–649. http://dx.doi.org/10.1007/s11606-005-0112-6 16050862 10.1111/j.1525-1497.2005.0105.x PMC1490157

[CIT0003] Angeli F, Sardone M, Angeli E, et al. Validation of the A&D wrist-cuff UB-511 (UB-512) device for self-measurement of blood pressure. Blood Press Monit. 2006;11(6):349–354. http://dx.doi.org/10.1097/01.mbp.0000217995.12215.f2 17106320 10.1097/01.mbp.0000217995.12215.f2

[CIT0004] Seedat YK, Croasdale MA, Milne FJ, et al. South African hypertension guideline 2006. S Afr Med J. 2006;96(4 Pt 2):337–362. 16670808

[CIT0005] Jones DW, Appel LJ, Sheps SG, et al. Measuring blood pressure accurately: New and persistent challenges. JAMA. 2003;289(8):1027–1030. http://dx.doi.org/10.1001/jama.289.8.1027 12597757 10.1001/jama.289.8.1027

[CIT0006] Schwartz AR, Haas DC, Gerin W, et al. Accurate measurement of blood pressure. JAMA. 2003;289(21):2792 http://dx.doi.org/10.1001/jama.289.21.2792-a 12783901 10.1001/jama.289.21.2792-a

[CIT0007] Alexis O Providing best practice in manual blood pressure measurement. Br J Nurs. 2009;18(7):410–415. http://dx.doi.org/10.12968/bjon.2009.18.7.41654 19373184 10.12968/bjon.2009.18.7.41654

[CIT0008] Wedgbury K, Valler-Jones T Measuring blood pressure using an automated sphygmomanometer. Br J Nurs. 2008;17(11):714–718. http://dx.doi.org/10.12968/bjon.2008.17.11.29642 18773588 10.12968/bjon.2008.17.11.29642

[CIT0009] Rose G Standardisation of observers in blood pressure measurement. Lancet. 1965;285(7387):673–674. http://dx.doi.org/10.1016/S0140-6736(65)91827-1 14258545 10.1016/s0140-6736(65)91827-1

[CIT0010] Petrie JC, O'Brien ET, Littler WA, et al. Recommendations on blood pressure measurement. Br Med J. 1986;293:611 http://dx.doi.org/10.1136/bmj.293.6547.611 3092951 10.1136/bmj.293.6547.611 PMC1341395

[CIT0011] Roubsanthisuk W, Wongsurin U, Saravich S, et al. Blood pressure determination by traditionally trained personnel is less reliable and tends to underestimate the severity of moderate to severe hypertension. Blood Press Monit. 2007;12(2):61–68. http://dx.doi.org/10.1097/MBP.0b013e3280b08317 17353647 10.1097/MBP.0b013e3280b08317

[CIT0012] McKay DW, Campbell NR, Parab LS, et al. Clinical assessment of blood pressure. J Hum Hypertens. 1990;4(6):639–645. 2096205

[CIT0013] Sala C, Santin E, Rescaldani M, et al. How long shall the patient rest before clinic blood pressure measurement? Am J Hypertens. 2009;19(7):713–717. http://dx.doi.org/10.1016/j.amjhyper.2005.08.021 16814126 10.1016/j.amjhyper.2005.08.021

[CIT0014] Jhalani J, Goyal T, Clemow L, et al. Anxiety and outcome expectations predict the white-coat effect. Blood Press Monit. 2005;10(6):317–319. http://dx.doi.org/10.1097/00126097-200512000-00006 16496447 10.1097/00126097-200512000-00006

[CIT0015] Bland M Frequently asked questions on the design and analysis of measurement studies: How can I decide the sample size for a study of agreement between two methods of measurement? [page on the Internet]. c2004 [cited 21 Sept. 2014]. Available from: http://www-users.york.ac.uk/∼mb55/meas/sizemeth.htm

[CIT0016] Bland M Regression towards mean, or, Why was Terminator III such a disappointment? [page on the Internet]. c2004 [cited 21 Sept. 2014]. Available from: http://www-users.york.ac.uk/∼mb55/talks/regmean.htm

[CIT0017] Villegas I, Arias IC, Botero A, et al. Evaluation of the technique used by health-care workers for taking blood pressure. Hypertension. 1995;26(6 Pt 2):1204–1206. http://dx.doi.org/10.1161/01.HYP.26.6.1204 7498997 10.1161/01.hyp.26.6.1204

[CIT0018] Myers MG, Godwin M, Dawes M, et al. Measurement of blood pressure in the office: Recognizing the problem and proposing the solution. Hypertension. 2010;55(2):195–200. http://dx.doi.org/10.1161/HYPERTENSIONAHA.109.141879 20038756 10.1161/HYPERTENSIONAHA.109.141879

[CIT0019] Campbell NR, McKay DW Accurate blood pressure measurement: Why does it matter? CMAJ. 1999;161(3):277–278. 10463050 PMC1230505

[CIT0020] Haynes RB, Sackett DL, Taylor DW, et al. Increased absenteeism from work after detection and labeling of hypertensive patients. N Engl J Med. 1978; 299(14):741–744. http://dx.doi.org/10.1056/NEJM197810052991403 692548 10.1056/NEJM197810052991403

[CIT0021] Joffres MR, Hamet P, Rabkin SW, et al. Prevalence, control and awareness of high blood pressure among Canadian adults. CMAJ. 1992;146(11):1997–2005. 1596849 PMC1490336

[CIT0022] Wonka F, Thümmler M, Schöppe A Clinical test of a blood pressure measurement device with a wrist cuff. Blood Press Monit. 1996;1(4):361–366. 10226260

[CIT0023] Hodgkinson J, Mant J, Martin U, et al. Relative effectiveness of clinic and home blood pressure monitoring compared with ambulatory blood pressure monitoring in diagnosis of hypertension: Systematic review. BMJ. 2011;342d3621. 10.1136/bmj.d3621 PMC3122300 21705406

[CIT0024] Bland JM, Altman DG Statistical methods for assessing agreement between two methods of clinical measurement. Lancet, 1986;i:307–310. http://dx.doi.org/10.1016/S0140-6736(86)90837-8 2868172

[CIT0025] Pollock MA, Jefferson SG, Kane JW, et al. Method comparison – a different approach. Ann Clin Biochem. 1992;29(Pt 5):556–560. http://dx.doi.org/10.1177/000456329202900512 1444169 10.1177/000456329202900512

